# Health care seeking behaviors of pregnant women in rural Amhara, Ethiopia: a qualitative study of perceptions of pregnant women, community members, and health care providers

**DOI:** 10.11604/pamj.2023.45.142.39771

**Published:** 2023-07-25

**Authors:** Fisseha Shiferie, Firehiwot Workneh Abate, Tigest Shifraw, Michelle Eglovitch, Hanna Amanuel, Grace J Chan, Sheila Isanaka, Amare Worku Tadesse, Alemayehu Worku, Anne CC Lee, Yemane Berhane

**Affiliations:** 1 Addis Continental Institute of Public Health, Addis Ababa, Ethiopia,; 2 Project HOPE Ethiopia Country Office, Addis Ababa, Ethiopia,; 3 Department of Pediatric Newborn Medicine, Brigham and Women´s Hospital, Harvard Medical School, 75 Francis St, Boston, Massachusetts 02115, United States,; 4 The Global AIM Lab, Brigham and Women´s Hospital, Department of Newborn Medicine, Building B, Suite 502, 45 Francis Street, Boston, Massachusetts 02115, United States,; 5 Department of Epidemiology, Harvard T.H. Chan School of Public Health, Boston, Massachusetts, United States,; 6 Departments of Nutrition and Global Health and Population, Harvard T.H. Chan School of Public Health, Boston, Massachusetts, United States,; 7 Infectious Disease Epidemiology, London School of Hygiene and Tropical Medicine, London, United Kingdom

**Keywords:** Health care seeking, antenatal care, rural Amhara, pregnant women

## Abstract

**Introduction:**

in Ethiopia, increasing access to basic antenatal and neonatal health services may improve maternal and newborn survival. This study examined perceptions regarding antenatal health seeking behaviors from pregnant women, their families, community members, and health care providers in rural Amhara, Ethiopia.

**Methods:**

the study was conducted in four rural districts of the Amhara region of Ethiopia. A total of forty participants who were living and working within the catchment areas of the selected health centres were interviewed from October 3^rd^ through October 14^th^, 2018. A phenomenological qualitative study design was used to understand participants’ perceptions and experiences about pregnant women's health care seeking behaviors.

**Results:**

early disclosure of pregnancy status was not common in the study area. However, the data from the present study further provided new information, suggesting that some women did disclose their pregnancy status early but preferentially only to their partners and close relatives. Most women did not seek care unless sick or experienced new discomfort or pain. Some reasons for the low utilization of available antenatal services include long distance to health facilities, lack of transportation, difficult topography, and discomfort with male providers.

**Conclusion:**

despite the rapid expansion of health posts and deployment of health extension workers since 2003, there are still critical barriers to accessing facility-based care that limit women’s health care seeking practices.

## Introduction

Preventable maternal and child morbidity and mortality are major challenges to health in low- and middle-income countries. Preventable maternal mortality is, on average, ten times higher in low- and middle-income countries than in high-income countries [1]. The major complications that account for 80% of maternal deaths during pregnancy include severe bleeding, infections, high blood pressure, obstructed labour, and unsafe abortion [2]. In Ethiopia, as in other low- and middle-income countries, there are high rates of maternal and newborn mortality that could be prevented by access to basic antenatal-neonatal health services. The high mortality rate in Ethiopia may be associated with the low utilization of maternal healthcare services. The 2016 Ethiopian Demography and Health Survey (EDHS) showed that the national antenatal care (ANC) service utilization, defined as four visits in pregnancy, was only 62% [3]. Healthcare service utilization has not significantly improved since the national survey in 2016, as shown in the recent study revealing that utilization of ANC services in Ethiopia still stood at 63.77% in 2019. However, it varied across regions; the highest being in Oromia (85.2%) and the lowest in the Amhara region (32.3%) [4].

Studies have shown that inadequate access to information about complications and danger signs could contribute to the low utilization of maternal healthcare services [2,5-7]. While a study done in the west Amhara region of Ethiopia indicated low satisfaction of clients regarding healthcare service provision as a major reason for reporting late to first ANC services [8,9], the 2016 survey by EDHS and another study done in Jimma zone, southwest Ethiopia, have shown that only 19-20% of pregnant women had their first antenatal care during the first trimester [3,10].

Women also may believe there are no advantages in attending ANC during the first trimester as ANC is perceived primarily as curative rather than preventive [11]. According to a study done in Southern Mozambique, other factors that could impede women from accessing maternal health services promptly include societal discouragement from revealing pregnancies early in gestation, complex and delayed decision-making, poor transport infrastructure, and fear of mistreatment at health facilities [12,13]. Several studies have been carried out in Ethiopia and sub-Saharan Africa about pregnant women´s utilization of healthcare services. The main participants in these studies were pregnant women themselves. The perspectives of community members and healthcare providers on pregnant women´s health care seeking behaviors have been under-explored. This qualitative study aimed to examine perceptions regarding antenatal health seeking behaviors from pregnant women, their families, community members, and health care providers in rural Amhara, Ethiopia.

## Methods

**Study site:** the study was conducted in four rural districts of the Amhara region of Ethiopia. The districts were chosen due to the high rates of maternal undernutrition and high ANC patient flow. South and North Achefer districts from the West Gojjam zone and Dera and Libokemkem districts from the South Gondar zone were chosen as potential sites for the Enhancing Nutrition and Antenatal Infection Treatment (ENAT) study (ISRCTN15116516)). One health centre was selected from each district, yielding a total of four health centres (Lalibela, Liben, Anbessamie, and Yifag health centres). Health posts within the catchment of these health centres were also included in the study. Interviews were conducted at the health centres, and for the health care providers, the interviews were conducted in the Maternal and Child Health (MCH) unit of health centres from October 3^rd^ through October 14^th^, 2018.

**Study participants:** a total of forty participants who were living and working within the catchment areas of the selected health centres (Lalibela, Anbessamie, Yifag, and Liben) and health posts in the South Gondar and West Gojjam zones took part in this study. Participants included sixteen pregnant women, twelve health care providers (midwives, nurses, and health extension workers (HEWs)), and twelve community members. Before commencing the interview, written consent was obtained from all participants. A purposeful sampling technique was used to select participants. Healthcare providers working in the maternal and child health department and pregnant women and community members who visited the study health centres at the time of data collection were included in this study. Data collection was stopped when data saturation was perceived to be reached, i.e. when no new information was elicited by consenting and interviewing more participants.

**Study design and data collection:** phenomenological qualitative study design was used to understand participants´ perceptions and experiences about pregnant women's health care seeking behaviors. In-depth interviews (IDIs) were conducted as the study aimed to elicit detailed individual experiences and perceptions of pregnant women, community members, and healthcare providers regarding pregnant women´s healthcare-seeking behaviors. The interviews were conducted in Amharic (the local language in the study area) in a private location within the selected health centres. Interviews were tape-recorded after obtaining written consent. An interview guide designed to explore the perceptions and experiences of pregnant women's health care-seeking behaviors among participants was developed in English, translated into Amharic, and then pretested and revised. Probing questions were included in the interview guide in case the participants´ responses were superficial, or the answers were conflicting. Interviews took between 40 and 70 minutes to complete. Experienced research assistants (RAs) with training in public health or social sciences and relevant experience in qualitative data collection techniques and who were fluent in the local language were recruited to interview participants. Research assistants attended a three-day training session in Addis Ababa on IDI data collection techniques. They conducted interviews in pairs, where one conducted the interview, and the other RA took notes. Researchers did daily supervision and daily debriefing sessions were conducted at the end of each day to troubleshoot daily challenges and encounters.

**Data analysis:** a qualitative content analysis approach was chosen to analyze the data. All interviews were transcribed verbatim in Amharic and translated into English. Transcripts were manually coded, and themes were generated. Themes were reviewed to make sure that they accurately represented the data. Some of the themes were split up; others were combined or discarded. Daily summaries and field notes were also reviewed as part of the analysis and used to inform the selection of final themes.

**Ethics approval and consent to participate:** the Ethical Review Committees/Institutional Review Boards of Addis Continental Institute of Public Health and Partners Health Care/Mass General Brigham approved the formative study protocol. All participants in this study provided informed written consent before being interviewed.

**Funding:** this study was funded by Bill and Melinda Gates Foundation (OPP1184363).

## Results

**Figure 1:**
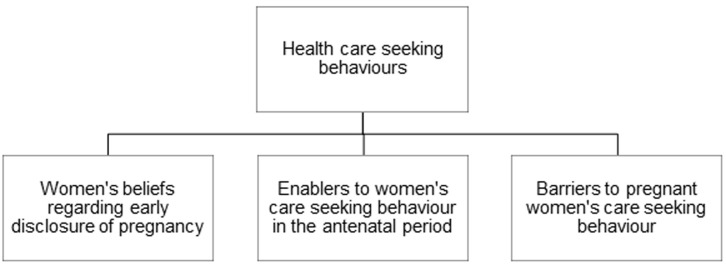
the three major themes that emerged from the in-depth interviews

In-depth interviews were conducted among 40 participants, including pregnant women (n = 16), community members (n = 12), and healthcare providers (n = 12). Three major themes emerged from the in-depth interviews: perceptions of women´s beliefs regarding early disclosure of pregnancy, enablers to women´s care-seeking in the antenatal period, and barriers to care-seeking (Figure 1).

### Beliefs regarding early disclosure of pregnancy

**Pregnant women:** pregnant women participants relied on different signs to confirm whether they had conceived or not. Missing periods, tiredness, and lack of appetite were among the most common signs. *I first knew that I was pregnant when my menses stopped. Other signs that have helped me confirm my pregnancy were tiredness and lack of appetite. However, I didn´t tell anybody about my pregnancy, although I knew I was pregnant*, [woman 1, Dera woreda]. Apart from missing periods to confirm pregnancy, a few women also considered conception when they felt the movement of the foetus in their uterus. *I first knew that I was pregnant after I felt the movement of the foetus inside the womb*, [woman 2, Libokemkem woreda]. Early disclosure of pregnancy status was not common in the study area. Women did not usually disclose pregnancy until reaching the late second trimester. However, if they did disclose early, they usually did so with their close relatives. *I have disclosed my pregnancy to my husband, mother, sister, and mother-in-law*, [woman 1, North Achefer woreda]. *I had more than 4 ANC visits during this pregnancy. I knew my pregnancy early (at one month) because of loss of appetite, nausea, and menses. I announced this to my husband immediately after medical confirmation. In rural areas like ours, women are afraid of announcing pregnancy to their husbands, but in urban areas, they do. Announcing pregnancy early helps get care and support at home*, [woman 2, South Achefer woreda].

Most women did not want to disclose despite knowing they would benefit from early disclosure of pregnancy to community members and health care professionals. Some reasons for not disclosing pregnancy included fear of spontaneous abortion or miscarriage and cultural beliefs about miscarriages. *It is uncommon to disclose pregnancy status to people, especially in its early stage. We do this not because we do not know the benefits but simply because it is a social norm. Our community believes that if we disclose a pregnancy in its early stage, it is highly likely that miscarriage will occur*, [woman 4, North Achefer woreda]. *I found out about my pregnancy at 18 weeks. Many women in my area, including myself doubt our pregnancy until the quickening. I did not yet announce pregnancy because it is shameful. I do not believe telling the community about it has any benefit. Some women do not know about their pregnancy until their belly becomes prominent and visible*, [woman 3, Libokemkem woreda]. *This is my second visit for this pregnancy, and I attended four antenatal care visits during my first pregnancy. Most women visit health centres for the first time, usually after four months of pregnancy*, [woman 3, South Achefer woreda].

**Community members:** there were no major differences between pregnant women´s perceptions and community member participants regarding women´s disclosure of their pregnancy status. There was an agreement between both groups on the factors that affect early disclosure of pregnancy. *Women knew that they were pregnant when they missed menses. Some women might go to the facility to get tested to confirm their pregnancy if a health facility is close to their home*, [community member 1, Libokemkem Woreda]. *Women do not want to disclose their pregnancy until it is visible. Fear of miscarriage and shyness are among the reasons to keep pregnancy secret*, [community member 1, South Achefer Woreda].

Participants from the community also mentioned the role of health extension workers (HEWs) in teaching women to disclose pregnancy earlier. In their training, HEWs were more focused on the importance of early disclosure of pregnancy and the negative impact of associated beliefs. *The most important indicator of pregnancy is missed menses. In our village, women disclose their pregnancy to close family members and neighbours. Nowadays, due to the continuous health promotion and education by health extension workers, more women have started announcing their pregnancy as early as possible*, [community member 2, Dera Woreda]. *Currently, the belief associated with early disclosure of pregnancy has started decreasing. My wife used to be the most conservative and hesitant to visit a health care facility but now her attitude about announcing and attending ANC has completely changed*, [community member 2, North Achefer woreda].

### Enablers of pregnant women´s care-seeking behaviors in the antenatal period

**Pregnant women:** most women visited health centres for illness, new discomfort, or pain. *I knew I was pregnant because of my discomfort around my abdomen. Many pregnant women in my area deliver without attending antenatal care because they do not want to go to the facility unless they are sick- due to a lack of knowledge and awareness*, [woman 6, North Achefer woreda]. *This is my 2^nd^ antenatal care visits for my 2^nd^ pregnancy. I also attended ANC for my first pregnancy. During my first pregnancy, I knew I was pregnant after I came to the facility because I had a bleeding and vaginal discharge*, [woman 5, Dera Woreda]. While rare, some women visited facilities because they wanted to get pregnancy testing or screenings. *I knew that I was pregnant after I got a pregnancy test. I then started attending ANC 6-weeks after LMP because of headaches and nausea. I was treated for UTI as well. I will continue my ANC follow-up until nine months*, [woman 5, South Achefer woreda]. Participants also noted that their awareness of the importance of attending antenatal care visits had grown over time. *This is my fourth pregnancy and my fourth ANC visit for this pregnancy. I did not attend ANC for the first three pregnancies. But now, I am well aware of the importance of attending ANC and delivering in a facility. I have also learned that ANC attendance reduces sickness and death during pregnancy and delivery. Despite these benefits, I do not still believe in the importance of early disclosure of pregnancy status to others- visual signs are enough*, [woman 4, Dera Woreda].

Pregnant women considered different factors before deciding whether to seek care. One of our participants mentioned service quality as a factor in seeking care. *For me, service quality is the main factor in deciding whether to seek care during pregnancy and delivery*, [woman 6, North Achefer woreda]. The majority of participants knew the benefits of either attending antenatal care or delivering in a health facility, or both. Some of the benefits they recognized included obtaining iron folate tablets and hygiene and nutritional counseling. *I have received iron folate tablets, and deworming medications and got immunized. I also want to deliver in a health facility to be supported by trained healthcare providers during labour/delivery and get medication if bleeding occurs. Other services I usually get during my antenatal care visits include advice on personal hygiene, the types of food I should eat, and the types of activities I should perform during pregnancy*, [Woman 6, Dera woreda]. Although women reported it was costly to get antenatal care services in private clinics, participants also said they preferred private clinics over government facilities. That was mainly due to the shorter waiting time and perceptions of relatively better-quality care (safe, effective, and efficient, according to participants). *I started ANC at the private clinic but switched to a government facility because the cost of delivery in a private clinic is very expensive. If I should deliver at a private facility, it would be mainly for the shorter waiting times and better-quality care*, [woman 8, North Achefer woreda].

**Community members:** health extension workers worked with neighborhood administrators in identifying pregnant women and providing health education to them. As a result, a considerable number of women were attending antenatal care. However, a penalty (for example: a money penalty) would follow if a pregnant woman was found not attending ANC. *An increasing number of women are attending antenatal care services. If a pregnant woman is found not attending antenatal care and delivered at home, she will be fined by the kebele administrators. Identifying such women is the administrators´ top priority in our kebele. Health education is regularly provided to the community during public holidays and saint days. Women usually start antenatal care at four months of their pregnancy*, [community member 5, Libokemkem woreda]. *Because of health extension workers´ hard work, many women in my area attend antenatal care. In doing so, I think they have benefited from the advice from the health care providers*, [community member 4, North Achefer woreda].

Participants also shared their stories regarding the health consequences of failing to seek care during pregnancy. Stillbirths, and losing wives and children were some of the problems they encountered because their wives delivered at home. These same community participants are now engaged in letting others know about the benefits of seeking health care. *After losing my first wife, who delivered at home, I kept advising people to attend their antenatal care and deliver in health care facilities. Although we did everything that is locally acceptable and later took her to a health facility, it was too late to save her life*, [community member 6, South Achefer woreda]. *Four of my children had died because we had no information about antenatal care services by then. But had it been now, all of them would have survived*, [community member 4, Dera Woreda]. My wife had once delivered at home, and delivery was not successful. From then on, I advised her and others I know to deliver in a health facility, [community member 3, North Achefer woreda].

**Health care providers:** healthcare providers are described as having a positive relationship with women, motivating women to seek care in health facilities. Midwives, nurses, and health extension workers explained the kind of relationships pregnant women had with them in slightly different ways. *I have been working as a midwife for the last five years and it has been three years since I joined this health centre. I would say I have built a good relationship with pregnant women and other mothers who come to this health centre to receive various healthcare services. The trust and the respect they have for us are simply amazing. They always consider us as reliable sources for their information needs. We usually meet pregnant women when they come to health centres seeking services like antenatal, birth, and postnatal services. In addition, they also come in between appointments whenever they have any issues including illness. In all of these contacts, we always strive to provide services in the best possible way*, [Midwife, Libokemkem woreda]. Healthcare providers usually met with women in healthcare facilities or at women´s homes. Health extension workers spent much of their time in villages providing home-to-home services. This interaction of HEWs with women was believed to strengthen their relationship and helped women to seek care in health facilities. *Unlike other healthcare providers, our contact with women is not only limited around health facilities. We usually go out of health posts and provide services home-to-home. Monthly conferences, one-to-five meetings, and vaccinations are other platforms that bring us together. Due to this reason, it has now become very common to consider us as members of their families. During our daily home-to-home visits, they invite us for lunch and to have some coffee with them. Our relationship extends further and we get invited for weddings and other festivals. I think this shows the kind of intimacy we have with women*, [health extension worker, South Achefer woreda].

It was not very common for nurses and midwives to provide healthcare services outside healthcare institutions. However, on some occasions, such as holidays, healthcare staff went to villages and educated the community on various topics in smaller groups around their homes or in big gatherings around churches. In terms of strengthening their relationship with women, out-of-facility meetings were found to be more productive. One of the nurses explained the reason as follows. *If not very often, we also go to different kebeles where we provide health education to the community, including pregnant women. This mainly happens during saint days like St. Michael, St. Mary, St. Gabriel, etc. In this community, Orthodox Tewahdo Christians have a special kind of respect for these Saints. One way of showing this respect is by calling their close relatives to their homes so that they can have some drinks and food together. Considered one of their relatives, I have been invited to their homes many times. In my opinion, this frequent contact and our friendly approach have shaped our relationship to look more like a family than a service provider and service recipient*, [nurse, North Achefer woreda].

### Barriers to care-seeking in the antenatal period

**Pregnant women:** various factors prevented women from seeking care. Access to health centres (including distance and transportation) and season were among the obstacles. *Distance remains the biggest challenge to regularly visit health facilities, especially during the rainy season*, [woman 4, North Achefer woreda]. *Despite other factors, the distance between my home and a health facility is the only thing I consider when deciding whether to seek care and where to get health care services*, [woman 7, Libokemkem woreda]. *Distance is the primary factor I consider whether I should get health care services or not. Despite this, I believe in getting antenatal care services and institutional delivery both for the safety of myself and my baby*, [woman 7, North Achefer woreda]. Apart from distance, women also mentioned the terrain that made health centres inaccessible. *It is not only the distance that hinders me from getting the services but also the difficult topography that made transportation inaccessible to ambulances, bajaj, and car. The community brings sick people to healthcare facilities using a locally made bed. I should travel around 2 hours to get the services I need*, [woman 5, Dera woreda].

**Community members:** unlike health care providers who have established good relationships with women, the disrespect and lack of compassion by some healthcare providers were alarming to some community members. *I am convinced that healthcare facilities are the right places to get any kind of health care, including antenatal care and delivery. However, I am very concerned about the capacity and ethics of some healthcare providers. There are healthcare providers who are disrespectful and not compassionate to women. Delivering in a healthcare facility benefits the mother-baby pair in many ways. It also avoids the harmful traditional practices that are performed at home, such as feeding the newborn with butter, washing the newborn with cold water*, etc. [community member 5, South Achefer woreda]. Community members also added that women preferred home to health facilities for delivery because they preferred personal privacy. *I believe that pregnant women should attend their antenatal care and deliver in health facilities. However, some still prefer to deliver at home because they do not want their private parts seen by healthcare providers*, [community member 3, Libokemkem woreda].

## Discussion

In this study, pregnant women's health care seeking behaviors were explored based on women´s, family members, and health care providers´ perceptions of early disclosure of pregnancy as well as enablers and barriers to women´s care-seeking in the antenatal period. According to the data generated from this study, early disclosure of pregnancy status was not common in the study area. Women do not usually disclose pregnancy until it is in the late second trimester. This might result in women´s late report for their first ANC or forgoing all ANC. The major reasons were fear of and perspectives on spontaneous abortions and miscarriages. Similar results were obtained in rural communities of southern Mozambique, where societal discouragement from revealing pregnancies early in gestation impeded women from timely accessing maternal health services [12]. A study conducted in Nigeria also reported a similar result regarding women´s early disclosure of their pregnancies. In that setting, it was believed that early disclosure might lead to miscarriage and other complications as women thought that supernatural forces had the potential to influence pregnancy outcomes. As a result, women avoided routine activities that could reveal pregnancy status, including antenatal care, and they might delay antenatal care until the seventh month of pregnancy [11]. The data from the present study further provided new information, suggesting that some women did disclose their pregnancy status early but preferentially only to their husbands and close relatives. Reports of limited disclosure are similar to results from qualitative studies conducted in Ghana, Kenya, and Malawi. In Ghana, women reported limited disclosure to avoid the embarrassment they would experience if they did not bring the pregnancy to term [14].

Results of this study showed that most women did not seek care unless they were sick or experienced new discomfort or pain. A study conducted in Ogun State in Nigeria [11] and in rural Haramaya district in eastern Ethiopia found similar results to the current study, which reported that women accessed the formal health care system only when they perceived they were at risk [15]. A study conducted in Pakistan is also consistent with the current study´s findings on when women usually seek care. According to this study, most women prefer to deliver at home when they perceive that delivery would be free of any complications. These are women who had received ANC from skilled professionals in the third trimester of their pregnancy, which enhanced their confidence about pregnancy and delivery to be free of complications [16].

Although Ethiopia has invested in its healthcare infrastructure by expanding health facilities and deploying health extension workers, the utilization of healthcare services by pregnant women remains among the lowest in Africa [17]. According to the data from this study, some women still choose to deliver at home. Despite the rapid expansion of health posts and deployment of health extension workers since 2003, there are still critical barriers to accessing facility-based care that limit women´s healthcare seeking behavior. Some reasons for the low utilization of available services include long distances to health facilities, lack of transportation, difficult topography, inadequate quality of care at the facilities, and discomfort with male providers. In Ethiopia, accessing health facilities, especially in the rainy season, is very difficult as flooding damages the quality of roads. Studies conducted in Ethiopia and other countries revealed similar results about women´s low utilization of health care services. Findings from Northwest Ethiopia revealed that the traveling time of respondents was significantly associated with the utilization of ANC service. Pregnant mothers who traveled less than one hour were eight times more likely to attend antenatal care than those who travel more than one hour [18]. A study done in Nepal mentioned cultural practices, shyness to be seen by men providers, and attitudes of health care providers to have influenced women´s decisions not to seek care [19]. Another study in Zimbabwe reported rude behavior of health care staff and poor quality of care for not seeking health care [20]. However, this finding contradicts a study conducted in other parts of Ethiopia, which reported high coverage of ANC service that might have been attributed to health promotion activities on maternal and child health issues by developmental nongovernmental organizations [10].

**Strengths and limitations of the study:** the strength of this study is that it assessed the perceptions of pregnant women´s families, community members, and healthcare providers in addition to the perceptions of pregnant women themselves regarding their health care seeking behaviors in rural Amhara. This study´s limitations include that the study was only limited to rural women's health care seeking behaviors, which may differ from those of women living in urban areas. The other limitation was that recall bias might have affected participants´ responses. The third limitation is that generalizability cannot be presumed due to the limited geographic scope and sample size of this study.

## Conclusion

Generally, despite the government´s commitment to increase access to antenatal care by rapidly expanding health posts and deploying health extension workers, there are still several barriers to women's health care seeking during pregnancy, including transportation challenges, quality of care provided in facilities, and hesitancy to disclose pregnancy. The present study showed that most women only seek care when they are sick or experience new discomfort or pain. In addition, women do not usually disclose pregnancy until it is the late second trimester, although there are some women who preferentially disclose their pregnancy statuses only to their husbands and close relatives as early as possible.

### What is known about this topic

- *There was low satisfaction of clients regarding healthcare service provision leading to late reporting to the first ANC services*.

### What this study adds

- *The present study revealed that out-of-facility meetings were found to be more productive in terms of strengthening the relationship between healthcare providers and women;*
- *Although early disclosure of pregnancy status was not common in the area, this study added to the existing literature that some women disclosed their pregnancy status early but preferentially only to their husbands and close relatives;*
- *The rapid expansion of health posts and deployment of health extension workers since 2003 to raise awareness of health issues did not bring the expected change in women’s health care seeking behavior*.
